# TIPE2 acts as a biomarker for GIST risk category and suppresses the viability and invasiveness of GIST cells

**DOI:** 10.1186/s13578-018-0261-z

**Published:** 2018-12-05

**Authors:** Zequn Li, Wei Zhang, Yi Li, Shougen Cao, Shanglong Liu, Liang Ning, Xuelong Jiao, Zimin Liu, Xiaoming Xing, Yujun Li, Yanbing Zhou

**Affiliations:** 1 Department of General Surgery, Affiliated Hospital of Qingdao University, 16# Jiangsu Road, Qingdao, Shandong People’s Republic of China; 2 Department of Emergency Medicine, Affiliated Hospital of Qingdao University, Qingdao, China; 3 Department of Emergency General Surgery, Affiliated Hospital of Qingdao University, Qingdao, China; 4 Department of Oncology, Affiliated Hospital of Qingdao University, Qingdao, China; 5 Department of Pathology, Affiliated Hospital of Qingdao University, Qingdao, China

**Keywords:** TIPE2, GIST, Risk category, Rac1

## Abstract

Evaluating the risk category of gastrointestinal stromal tumors (GISTs) is crucial for predicting prognosis and choosing treatment strategies, and tumor metastasis usually represent poor prognosis. Tumor necrosis factor-alpha-induced protein 8-like 2 (TIPE2) is a novel described tumor suppressor. In the present study, TIPE2 expression was detected using a total of 96 human GIST specimens by immunohistochemistry. The effect of TIPE2 on proliferation and invasiveness of GIST cells and its related mechanisms were explored in vitro. It was found that TIPE2 expression was gradually decreased in accordance with GIST risk grades and negatively associated with tumor size, mitotic count and risk category. Moreover, TIPE2 was identified as a biomarker for evaluating the risk grade of GIST. TIPE2 markedly suppressed the viability, colony formation, migration and invasion of GIST cells. Furthermore, TIPE2 induced apoptosis and suppressed MMP-9 expression of GIST cells by targeting Rac1. In conclusion, these results indicate that TIPE2 plays a pivotal role in the progression of GIST. TIPE2 serves as a promising biomarker for evaluating GIST risk grade and a potential target for treatment of GIST.

## Introduction

GISTs are mesenchymal neoplasms that arise in the gastrointestinal (GI) tract [1–3]. According to the modified NIH criteria, the distribution of risk for primary GIST is categorized as very low risk (15%), low risk (30%), intermediate risk (22%) and high risk (33%), which is determined by tumor mitotic rate and size, tumor site, and presence of tumor rupture [4]. Till now, surgery remains mainstay of treatment for GIST patients. More than half of the GIST patients ultimately develop local recurrence or distant metastasis, which often leads to fatal prognosis [1, 5–7]. Currently, the criteria for risk evaluation depend largely on clinicopathologic factors such as tumor size and mitotic rate, which cannot predict the risk of malignancy accurately [2, 8]. The detailed molecular mechanisms of this mesenchymal neoplasm are poorly understood. Therefore, discovering novel biomarkers and a better understanding of the detailed molecular mechanisms for the progression of GIST is urgently needed.

Tumor necrosis factor (TNF)-α-induced protein 8 (TNFAIP8) like 2 belongs to the TNFAIP8 family [9]. TIPE2 was initially identified as a negative immune regulator that is of vital importance in maintaining immune homeostasis [10, 11]. It has been demonstrated that TIPE2 is down-regulated in patients with infectious or autoimmune diseases [12–14]. Moreover, recent research found that TIPE2 was also involved in the development of a variety of tumors [15–18]. Our previous research found that by inhibiting the activity of Rac1, TIPE2 could suppress the tumorigenesis and development of tumor cells in HCC and NSCLC [15, 17–19].

Rac1 belongs to the Ras superfamily of small GTPases, research found that Rac1 overexpression is correlated with poor prognosis in a number of tumors [20, 21]. Rac1 plays a pivotal role in tumor development, especially in the invasiveness and metastasis of tumors [22, 23]. Several classical molecules associated with tumor invasiveness, such as MMPs and uPA, have been demonstrated to be the downstream effectors of Rac1 signaling [24]. Moreover, it has also been demonstrated that Rac1 is involved in regulating apoptosis of tumor cells [25, 26].

In the present study, we detected the expression of TIPE2 in GIST tissues and its clinical significance in evaluating GIST risk category. Moreover, in vivo experiments were conducted to explore the effect of TIPE2 on the malignant behaviors of GIST cells. Furthermore, we also investigated the related mechanisms for the aforementioned effect of TIPE2.

## Materials and methods

### Patients and tissues samples

A total of 96 human GIST specimens were collected from Department of General Surgery, Affiliated Hospital of Qingdao University. Immunohistochemical staining for CD117, CD34, DOG1, Ki-67 were conducted by the Department of Pathology to confirm the diagnosis of GIST and evaluate the risk category. According to the modified NIH criteria, the number of very low risk, low risk, intermediate risk and high risk were 22, 35, 18 and 21, respectively. All the experimental protocols were approved by the Ethics Committee of Qingdao University, China. Written informed consent was obtained from all subjects.

### Cell culture and transfection

GIST-T1 cells were cultured in DMEM medium (Gibco, CA, USA) supplemented with 10% inactivated fetal bovine serum (FBS) (Gibco, CA, USA) in a humidified cell incubator with an atmosphere of 5% CO_2_ at 37 °C. GIST-T1 cells were transfected with TIPE2 overexpression plasmid or Rac1 siRNA using Lipofectamine 2000 according to the manufacturer’s protocols (Invitrogen, Carlsbad, CA, USA). The mutant TIPE2 in which TIPE2 N-terminal lysine or arginine residues, Lys-15, Lys-16, and Arg-24 were replaced with glutamine or alanine was generated by PCR-based site-directed mutagenesis as previously described [27]. The Rac1 siRNA sequences purchased from Sigma-Aldrich (St. Louis, USA) were as follows: 5′-GCAAACAGAUGUGUUCUUA-3′, reverse 5′-UAAGAACACAUCUGUUUGC-3′. Sequences for nonspecific negative control: forward 5′-UUCUCCGAACGUGUCACGUTT-3′, reverse 5′-ACGUGACACGUUCGGAGAATT-3′. NSC23766 (Calbiochem, San Diego, USA), a specific Rac1 inhibitor, was used to inhibit Rac1 activity in some experiments.

### RNA isolation and real-time quantitative PCR

Total RNAs were extracted from transfected GIST-T1 cells using TRIzol reagent (Invitrogen, Carlsbad, CA, USA) and were reverse-transcribed into cDNA using a ReverTra Ace qPCR Kit (Toyobo, Osaka, Japan). Real-time PCR was performed using an UltraSYBR Mixture (CWBIO). The sequences of the sense and antisense primers were as follows: TIPE2: 5′-ACTGAGTAAGATGGCGGGTCG-3′, and 5-TTCTGGCGAAAGCGGGTAG-3′; Rac1: 5′-ATGTCCGTGCAAAGTGGTATC-3′, and 5-CTCGGATCGCTTCGTCAAACA-3′; GAPDH: 5′-AACGGATTTGGTCGTATTGGG-3′, and 5′-CCTGGAAGATGGTGATGGGAT-3′. Relative gene expression levels were normalized to GAPDH as control.

### Immunohistochemistry (IHC)

IHC was performed using paraffin-embedded tissue sections. The sections were dewaxed and hydrated, followed by antigen retrieval (in 0.01 mol/L citrate buffer solution, pH 6.0, heated to boiling for 2–3 min in a stainless steel pressure cooker). Endogenous peroxidase was blocked using a 3% hydrogen peroxide solution. The section was incubated with the blocking goat serum for 15 min and then immunostained with rabbit antibody against TIPE2 (dilution 1:100, Abcam, UK) or mouse antibody against Rac1 (dilution 1:100, Abcam, UK) at 4 °C overnight. Secondary staining was performed with HRP-conjugated anti-rabbit or anti-mouse IgG using a MaxVision Kit and a 3,5-diaminobenzidine (DAB) peroxidase substrate kit (Maixin Co, Fuzhou, China). The sections were then counterstained with hematoxylin.

### Evaluation of immunohistochemical staining

Immunohistochemical staining was independently evaluated by two experienced pathologists in a blinded manner. Staining was semi-quantitatively scored based on both the staining intensity (0, negative; 1, very weak; 2, weak; 3, moderate; 4, strong) and the percentage of positively stained cells (0, 0%; 1, 1–25%; 2, 26–50%; 3, 51–75%; 4, 76–100%). Both scores for each specimen were then combined to obtain the final score (IHC sum scores) of TIPE2 expression. The cut-off point for the sum of the scores was defined as follows: 0–2, low expression; 3–8, high expression.

### Cell viability assay

A total number of 3000 GIST-T1 cells were seeded in 96-well plates in triplicate wells and cultured for the indicated times. Cell viability was evaluated using the Cell Counting Kit-8 (CCK8) (Beyotime, Haimen, China) assay according to the manufacturer’s instructions. The absorbance was determined at 450 nm.

### Colony formation assay

GIST-T1 cells were seeded in six-well plates at a density of 500 cells per well and every 3 days the medium was replaced. Two weeks later, cells were washed by PBS and fixed with methanol for 10 min, then stained with 1% crystal violet. Colonies that consisted of more than 50 cells were counted and calculated as a percentage of that to the control group.

### Transwell assays for cell migration and invasion

The migration and invasion capacities of GIST-T1 cells were analyzed in 24-well Boyden chambers with 8-μm pore size polycarbonate membranes (Costar, Acton, USA). For invasion assay, the membranes were pre-coated with 50 μg Matrigel (BD Biosciences, San Diego, USA) to simulate matrix barriers. Cells (5 × 10^5^/mL) were resuspended in 200 μL serum-free medium and placed in the upper chamber. The lower compartments were filled with 600 μL medium with 10% FBS. The cells left on the upper surface of the membrane were removed after incubation. While the cells on the lower surface were fixed with methanol for 10 min and then stained with crystal violet for 20 min. Stained cell counting was performed under a light microscope at 200× magnification.

### Caspase activity assay

Caspase activity assays were carried out using a caspase fluorescent assay kit according to the manufacturer’s instructions. In brief, GIST-T1 cells were homogenized on ice with lysis buffer. An aliquot of 50 μL of supernatants was incubated with an equal volume of the reaction buffer containing fluorescent substrate at 37 °C for 1 to 2 h. Enzymatic release of free fluorescent moiety was measured by a fluorometer.

### uPA and MMPs activity assays

A total of 2 × 10^5^ GIST-T1 cells were cultured in a six-well culture plate and then treated with mock plasmid, TIPE2 overexpression plasmid, mutant plasmid, siNC or siRac1 for 24 h. The levels of secreted uPA, MMP-2 and MMP-9 in the culture supernatant were determined using an enzyme-linked immunosorbent assay (ELISA) following the manufacturer’s ELISA kit guidelines (R&D).

### Statistical analysis

A Chi square test of cross-tabulations and Fisher’s exact test were used to determine the associations between TIPE2 expression and clinicopathological parameters. Quantitative data are presented as the mean ± SD. The statistical significance was determined by two-tailed paired Student’s t-test between two groups, and paired comparison between multiple groups or for different time points was conducted. Receiver operating characteristic (ROC) curve analysis was performed to assess the diagnostic value of TIPE2 in GIST. All statistical analysis was performed using SPSS 18.0 software (SPSS Inc., Chicago, USA), and a *P* value < 0.05 was considered statistically significant.

## Results

### The expression of TIPE2 was gradually decreased in accordance with GIST risk grades

**Fig. 1:**
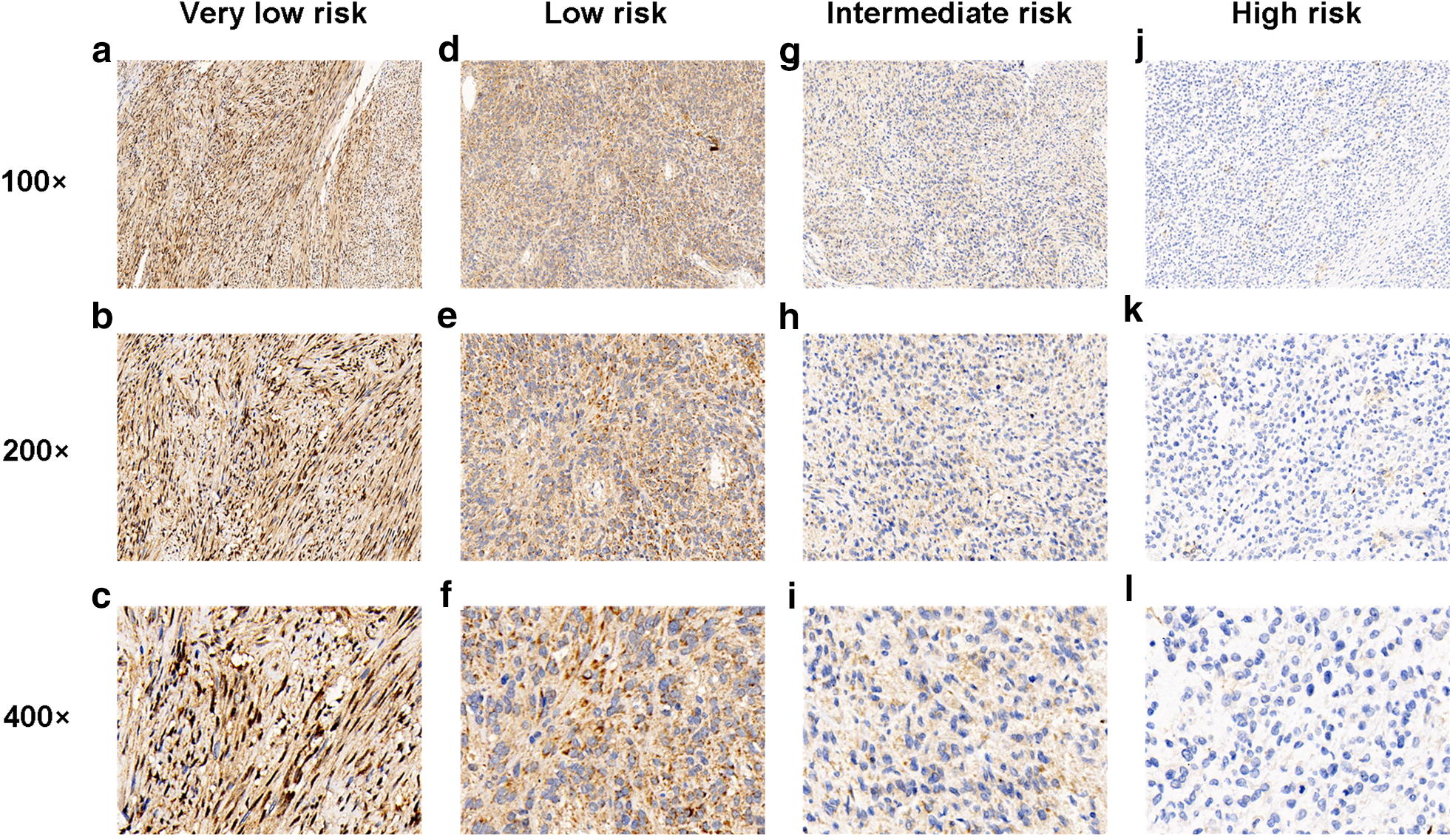
The expression of TIPE2 was gradually down-regulated in accordance with GIST risk categories. **a**–**c** Representative immunohistochemical staining showed high TIPE2 expression in very low risk GIST tissues. **d**–**f** Representative immunohistochemical staining showed moderate TIPE2 expression in low risk GIST tissues. **g**–**i** Representative immunohistochemical staining showed low TIPE2 expression in intermediate risk GIST tissues. **j**–**l** Representative immunohistochemical staining showed negative TIPE2 expression in high risk GIST tissues

IHC analysis was conducted using a total of 96 paraffin-embedded GIST samples with different risk categories. As shown in Fig. 1, TIPE2 was predominantly expressed in the cytoplasm of the tumor cells. Strong TIPE2 staining was observed in very low risk GIST tissues (Fig. 1a–c), while moderate TIPE2 expression was observed in low risk GIST tissues (Fig. 1d–f). TIPE2 expression was gradually decreased in accordance with GIST risk grades, as low and negative TIPE2 staining were shown in intermediate risk GIST tissues and high risk GIST tissues, respectively (Fig. 1g–i).

As shown in Table 1, although no significant association was shown between TIPE2 expression and Age (*P *= 0.2732), Gender (*P *= 0.8331) and Primary tumor site (*P *= 0.6585), TIPE2 expression was significantly associated with Tumor size (*P *= 0.0047), Mitotic count (*P *= 0.0005) and Risk category (*P *= 0.0006).

**Table 1:** Correlations between TIPE2 expression and clinicopathological characteristics in GIST tissues

Variable	Number	TIPE2 expression		P value
		Low	High	
Number of patients	96	34	62	
Age (years)				
< 60	59	18	41	0.2732
≥ 60	37	16	21	
Gender				
Male	49	18	31	0.8331
Female	47	16	31	
Tumor size (cm)				
≤ 2	20	3	17	*0.0047*
2–5	42	12	30	
> 5	34	19	15	
Mitotic count/50 HPFs				
≤ 5	82	23	59	*0.0005*
> 5	14	11	3	
Primary tumor site				
Gastric	61	23	38	0.6585
Non-gastric	35	11	24	
Risk category				
Very low risk	22	3	19	*0.0006*
Low risk	35	10	25	
Intermediate risk	18	6	12	
High risk	21	15	6	

Italic values represent significant value

### TIPE2 was a promising biomarker for evaluating the risk grade of GIST

**Fig. 2:**
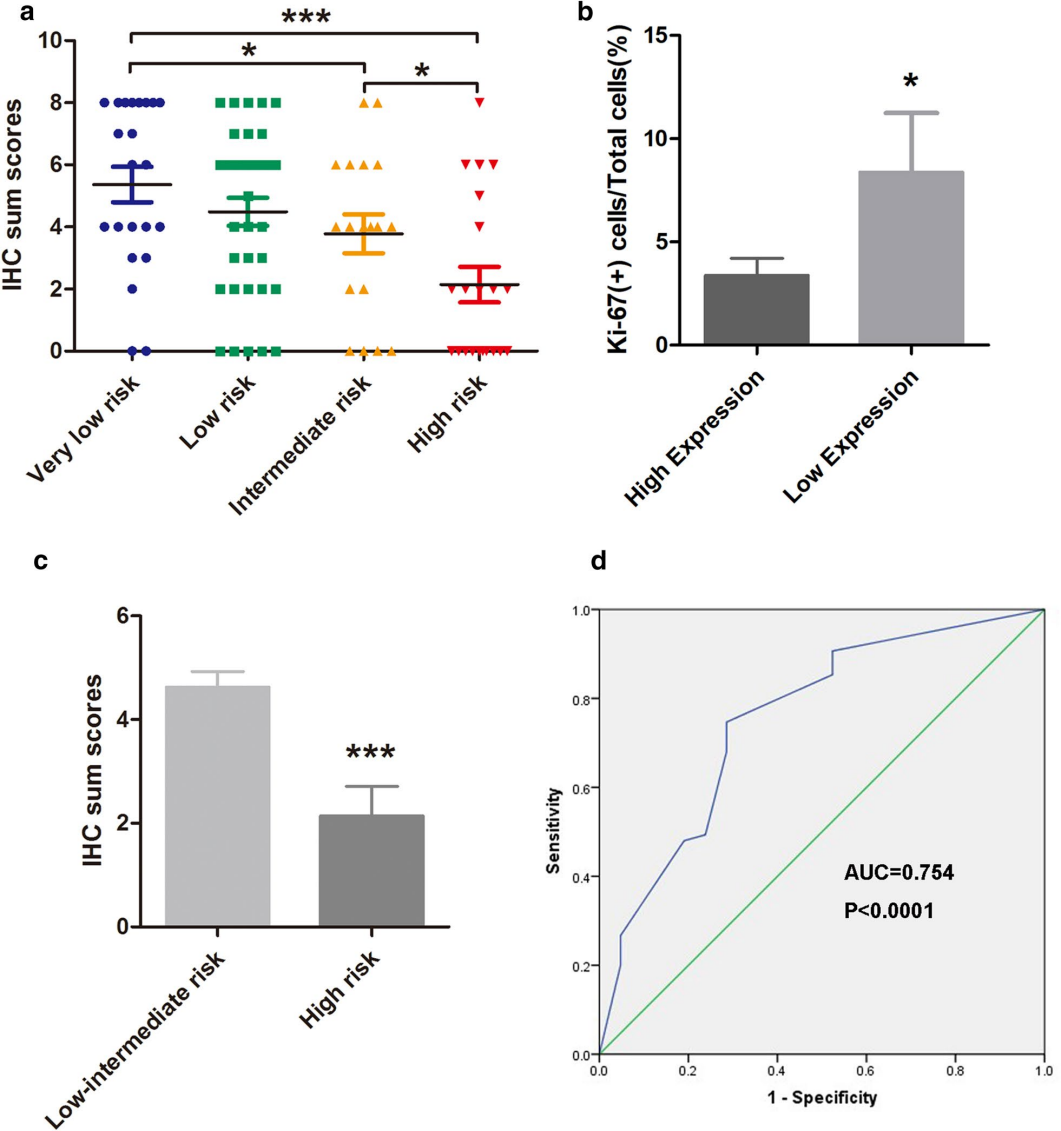
Statistical results of TIPE2 expression in GIST tissues and ROC curves curve to assess the diagnostic value of TIPE2 in GIST. **a** IHC sum scores showed that TIPE2 expression was gradually down-regulated in accordance with GIST risk grades. **b** IHC sum scores were used to assess the association between TIPE2 expression and the ratio of Ki-67 positive cells. **c** IHC sum scores showed a significant decrease of TIPE2 expression in high risk GIST tissues. **d** The ROC curves showed strong separation between low-intermediate risk GIST tissues and high risk GIST tissues, with an AUC of 0.754 (*P *< 0.001). *P < 0.05; ***P < 0.001

As shown in Fig. 2a, statistical analysis of IHC sum scores among groups confirmed that TIPE2 expression was significantly correlated with the risk category of GIST. Moreover, the ratio of Ki-67 positive cells in low TIPE2 expression GIST tissues was higher compared with that of high TIPE2 expression GIST tissues (Fig. 2b). As shown in Fig. 2c, TIPE2 expression was lower in high risk GIST group. To further determine the diagnostic value of TIPE2 expression in predicting the risk grade of GIST, receiver operator characteristic (ROC) curves were constructed and the area under the curve (AUC) was calculated. The ROC curves suggested that the AUC value for predicting the risk grade of GIST was 0.754 (Fig. 2d, CI (95%) 0.655–0.836, *P *< 0.0001), with an estimated sensitivity and specificity of 74.67% and 71.43% respectively (Table 2).

**Table 2:** Sensitivity, specificity, and positive and negative predictive values for evaluating GISTs risk category using TIPE2 IHC expression

IHC scores	Sensitivity	95% CI	Specificity	95% CI	PPV	95% CI	NPV	95% CI	Significance
≤ 2	74.67	63.3–84.0	71.43	47.8–88.7	90.3	80.1–96.4	44.1	27.2–62.1	High risk prediction

*PPV* positive predictive value, *NPV* negative predictive value

### TIPE2 suppressed the proliferation, colony formation, migration and invasion of GIST-T1 cells

**Fig. 3:**
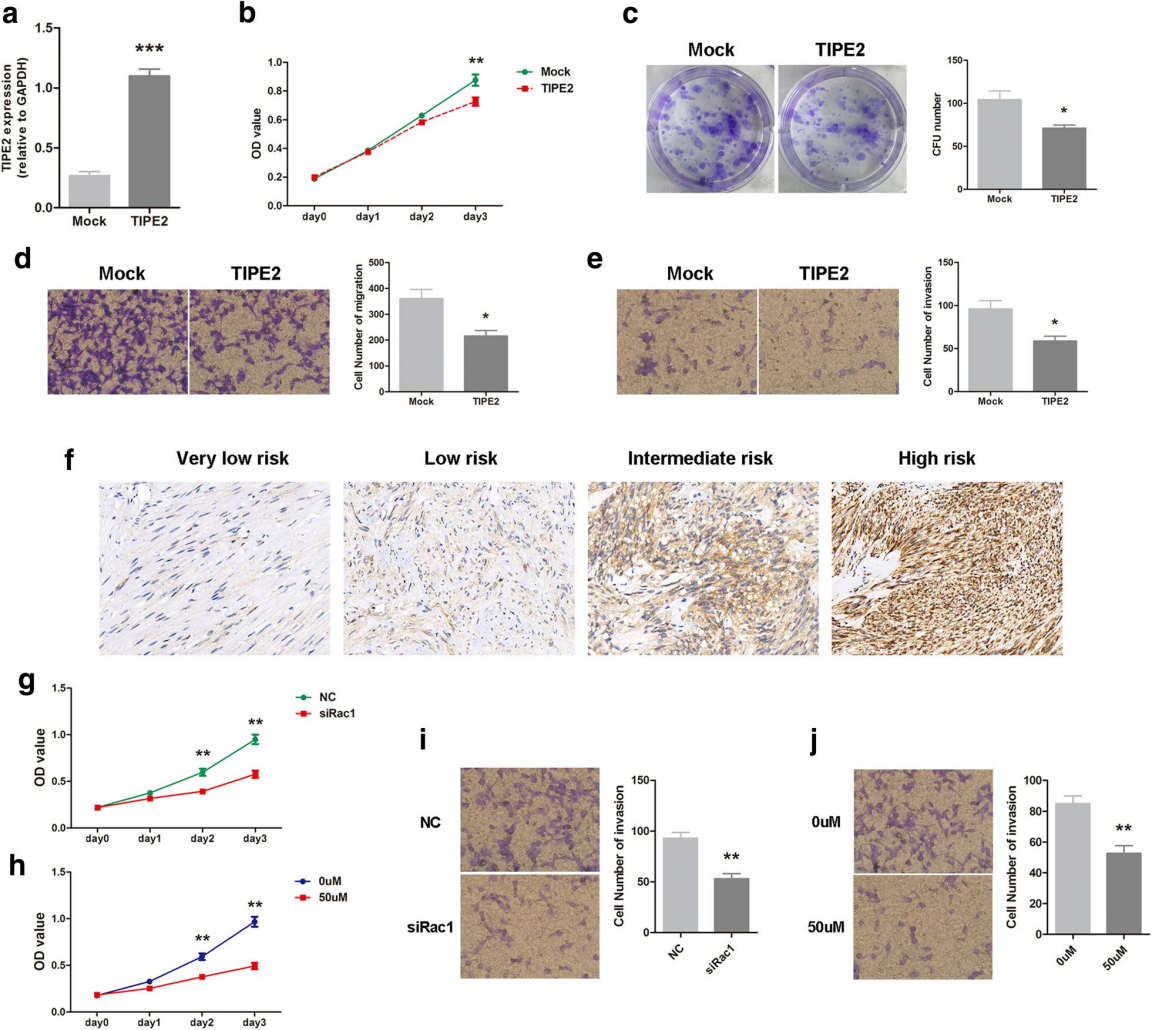
TIPE2 overexpression could suppress the proliferation, colony formation, migration and invasion of GIST-T1 cells by targeting Rac1. **a** Real-time PCR showed that TIPE2 expression was low in GIST-T1 cells and TIPE2 expression was up-regulated after transfection of TIPE2 overexpression plasmid. **b** CCK8 assays revealed that TIPE2 overexpression could suppress the viability of GIST-T1 cells. **c** After TIPE2 overexpression, colony formation assays were conducted to evaluated the colony formation capacity of GIST-T1 cells. **d**, **e** GIST-T1 cells transfected with the TIPE2 overexpression plasmid were used for Transwell migration and invasion assays. **f** Representative immunohistochemical staining showed that Rac1 expression was gradually up-regulated in accordance with GIST risk grades. **g** CCK8 assay was conducted to evaluate the viability of GIST-T1 cells after transfection with Rac1 siRNA. **h** CCK8 assay was conducted to evaluate the viability of GIST-T1 cells after treatment of NSC23766, a specific Rac1 inhibitor. **i** Transwell invasion assay revealed that Rac1 silencing decreased the invasiveness of GIST cells. **j** Transwell invasion assay was conducted to evaluate the invasiveness of GIST cells after treatment of NSC23766. **P < 0.01. Data represent the mean ± SD of three independent experiments. *P < 0.05; **P < 0.01; ***P < 0.001

Then the effect of TIPE2 on the malignant behaviors of GIST cells was explored in vitro. It was found that TIPE2 expression was low in GIST-T1 cells, so the TIPE2 overexpression plasmid was used. As shown in Fig. 3a, TIPE2 expression was up-regulated after transfection. CCK8 assays were used to evaluate the effects of TIPE2 on the viability of GIST cells, which revealed that TIPE2 overexpression suppressed GIST cell viability (Fig. 3b). Then colony formation assays demonstrated that TIPE2 also suppressed the colony formation capacity of GIST cells (Fig. 3c). Moreover, we performed Transwell migration and invasion assays in GIST-T1 cells after TIPE2 overexpression. As TIPE2 overexpression had no significant effect on cell viability within 24 h, which eliminated the potential confounding influence of TIPE2 induced suppression of cell proliferation on cell migration and invasion. Then results showed that TIPE2 overexpression could suppress both the migration and invasion capacities of GIST cells (Fig. 3d, e).

### Rac1 was involved in the viability and invasiveness of GIST cells

In order to investigate whether TIPE2 functions through targeting Rac1 in GIST, first we investigated the role of Rac1 in GIST. The expression of Rac1 in GIST tissues was detected using IHC. Results showed that Rac1 expression was gradually up-regulated in accordance with GIST risk grades, which was negatively associated with that of TIPE2 expression (Fig. 3f). Then we investigated the effect of Rac1 on the viability and invasiveness of GIST cells using Rac1 siRNA and NSC23766, a specific Rac1 activity inhibitor. CCK8 assays demonstrated that both Rac1 silencing and Rac1 inhibitor could suppress the viability of GIST cells (Fig. 3g, h). Further Transwell invasion assays found that Rac1 was also involved in the invasiveness of GIST cells (Fig. 3i, j).

### TIPE2 suppressed the viability and invasiveness of GIST cells via targeting Rac1

**Fig. 4:**
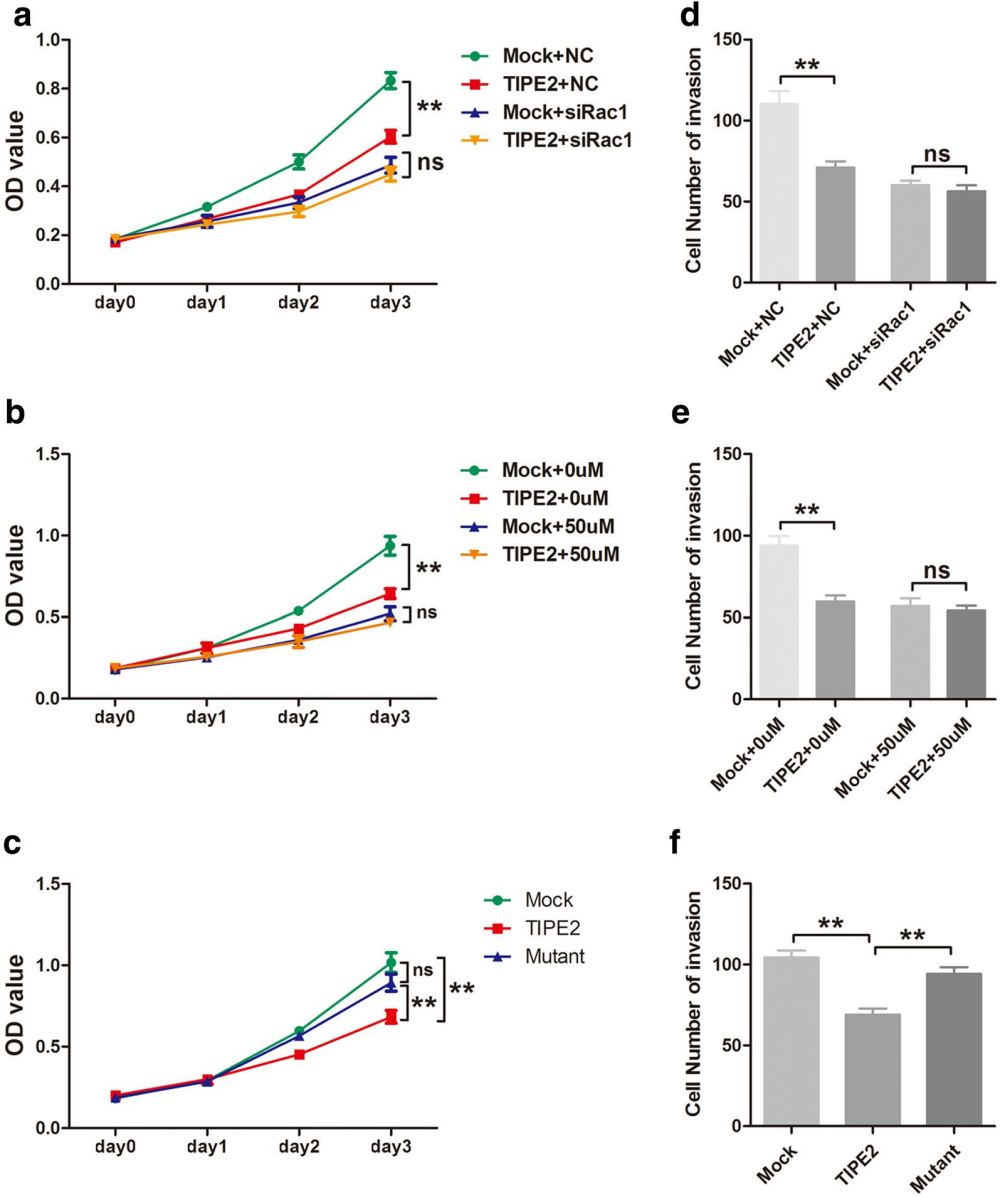
TIPE2 suppressed the proliferation and invasion of GIST cells by inhibiting Rac1. **a** After co-transfected with Rac1-specific siRNA and TIPE2 overexpression plasmid, GIST-T1 cells were applied to CCK8 assays. **b** After treatment with NSC23766, GIST-T1 cells were transfected with the TIPE2-overexpressing plasmid, and cell viability was measured by CCK8 assays. **c** GIST-T1 cells that transfected with mock, wild type TIPE2 and mutant TIPE2 plasmids were used for CCK8 assays. **d** GIST-T1 cells that co-transfected with Rac1-specific siRNA and TIPE2 overexpression plasmid were applied to Transwell invasion assays. **e** After treatment with NSC23766, GIST-T1 cells were transfected with the TIPE2-overexpressing plasmid, and cell invasiveness was measured by Transwell invasion assays. F. GIST-T1 cells that transfected with mock, wild type TIPE2 and mutant TIPE2 plasmids were used for Transwell invasion assays. **P < 0.01

To demonstrate whether TIPE2 functions through targeting Rac1 or not, the following experiments were performed. First, CCK8 assays were performed in GIST-T1 cells after co-transfected with TIPE2 overexpression plasmid and Rac1 siRNA. Results showed that Rac1 siRNA eliminated the inhibitory effect of TIPE2 on the viability of GIST-T1 cells (Fig. 4a). Moreover, GIST cells pretreated with NSC23766 also eliminated the effect of TIPE2 on cell viability (Fig. 4b). Further, the mutation of TIPE2 in sites which binds to Rac1, reversed the inhibitory effect of TIPE2 on cell viability (Fig. 4c). Similarly, both Rac1 siRNA and NSC23766 could eliminate the inhibitory effect of TIPE2 on the invasiveness of GIST-T1 cells, and mutant TIPE2 plasmid also reversed the effect of TIPE2 on cell invasion (Fig. 4d–f).

### TIPE2 induced apoptosis and decreased MMP-9 expression of GIST cells in Rac1 dependent manner

**Fig. 5:**
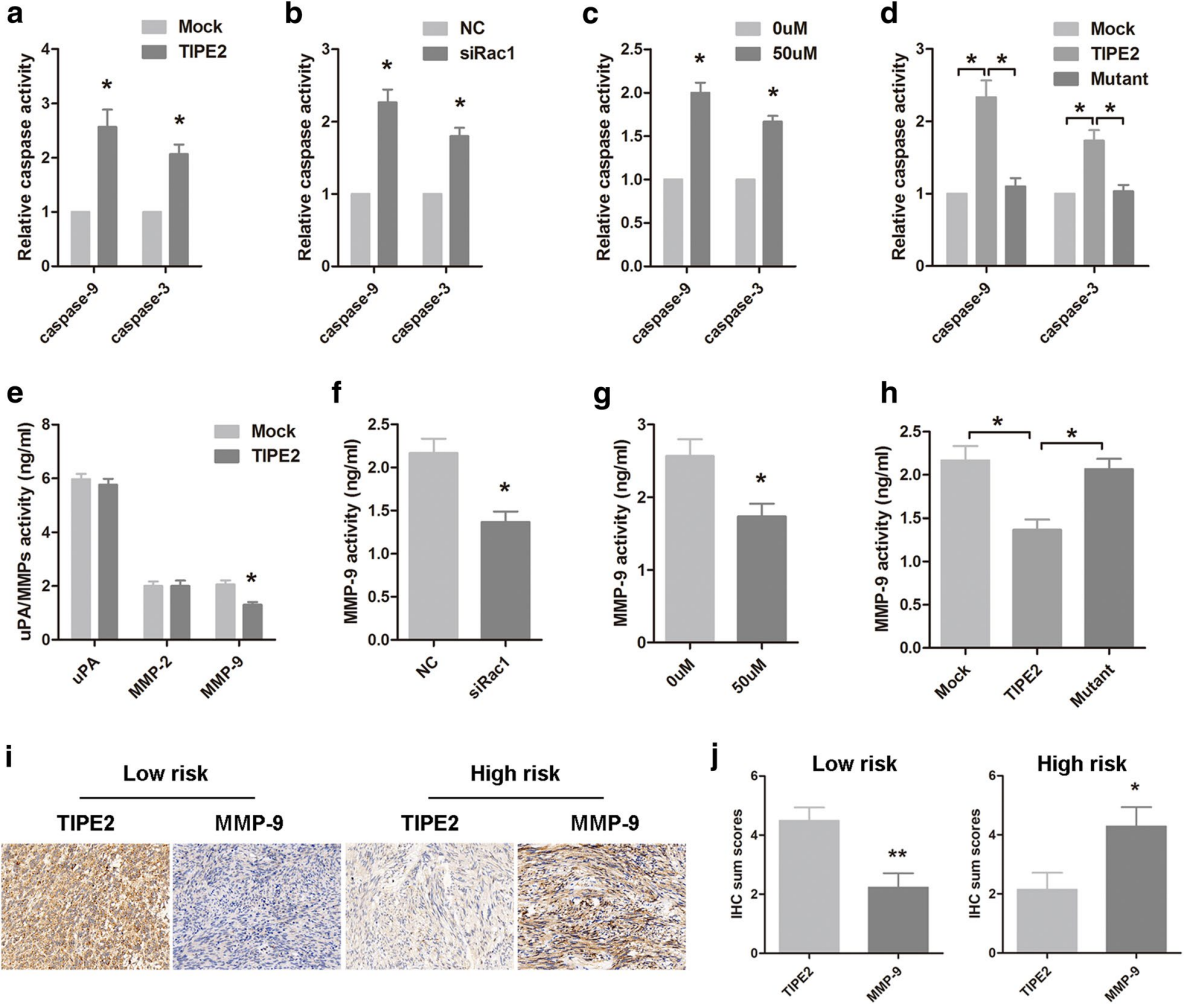
TIPE2 induced apoptosis and suppressed the expression of MMP9 in a Rac1-dependent manner. **a** Caspase-9/3 activity of GIST-T1 cells that transfected with TIPE2 overexpression plasmid were examined using the fluorescent caspase activity assay. **b** Caspase-9/3 activity of GIST-T1 cells that transfected with Rac1 siRNA were examined using the fluorescent caspase activity assay. **c** Caspase-9/3 activity of GIST-T1 cells that pretreated with NSC2366 were examined using the fluorescent caspase activity assay. **d** GIST-T1 cells that transfected with Mock, TIPE2 overexpression plasmid and TIPE2 mutant plasmid were applied to the fluorescent caspase activity assays. **e** The protein level of uPA, MMP-2 and MMP-9 were detected by ELISA in GIST-T1 cells transfected with TIPE2 overexpression plasmid. **f** The protein level of MMP-9 was detected by ELISA in GIST-T1 cells transfected with Rac1 siRNA. G. The protein level of MMP-9 was examined by ELISA in GIST-T1 cells pretreated with NSC23766. H. GIST-T1 cells that transfected with Mock, TIPE2 overexpression plasmid and TIPE2 mutant plasmid were used for MMP-9 activity assays. *P < 0.05. **i** Representative immunohistochemical staining showed high TIPE2 expression and low MMP-9 expression in low risk GIST tissues, while the MMP-9 expression was high in high risk GIST tissues. **j** IHC sum scores showed low MMP-9 expression and high TIPE2 expression in low risk GIST tissues, while high MMP-9 expression and low TIPE2 expression in high risk GIST tissues

Previous research demonstrated that Rac1 is important in regulating apoptosis [25, 26]. To investigate whether TIPE2 suppressed the viability of GIST cells via inducing apoptosis, caspase-9 and caspase-3 activities of GIST-T1 cells were detected after TIPE2 overexpression. Results showed that TIPE2 increased caspase-9 and caspase-3 activity in GIST cells (Fig. 5a). To further explore the role of Rac1 in TIPE2 induced apoptosis, Rac1 siRNA and inhibitor were used. It was found that both Rac1 silencing and NSC23766 could increase the activities of caspase-9 and caspase-3 (Fig. 5b, c). More importantly, mutant TIPE2 reversed the effect of TIPE2 on apoptosis (Fig. 5d), indicating that TIPE2 induced apoptosis of GIST cells via inhibiting Rac1.

It is evident that Rac1 plays a pivotal role in regulating tumor invasiveness, and uPA and MMPs have been demonstrated to be the downstream effectors of Rac1 signaling [28, 29]. Then the effect of TIPE2 on the expression of uPA, MMP-2 and MMP-9 was detected. It was found that TIPE2 overexpression decreased the expression of MMP-9 in GIST cells, while no significant changes were shown on uPA and MMP-2 expression (Fig. 5e). Moreover, both Rac1 siRNA and Rac1 inhibitor also suppressed MMP-9 expression (Fig. 5f, g). Furthermore, the mutation of TIPE2 in sites which binds to Rac1 reversed the inhibitory effect of TIPE2 on MMP-9 expression (Fig. 5h). In addition, the expression of MMP-9 in GIST tissues was negatively associated with TIPE2 expression (Fig. 5i, j).

## Discussion

The notion of GISTs was preliminarily introduced in the 1980s, but these neoplasms were not widely recognized until the start of the 21st century, when the molecular biology and genetic diagnosis were generalized [30]. Nowadays, the National Institutes of Health (NIH) consensus criteria, Armed Forces Institute of Pathology (AFIP) criteria, and modified NIH criteria are frequently used to estimate the risk category of GISTs [4, 8, 31]. Although the prognostic value of these schemes has been validated, more confounding factors that may improve the evaluation of GIST risk grade are still needed [32, 33].

TIPE2 belongs to the TIPE family and was preliminarily identified as an immune negative regulator. Recently, TIPE2 has been found to be involved in various of epithelium derived carcinomas [18, 19, 29, 34]. However, the expression and roles of TIPE2 in GIST, a special kind of mesenchyme originated neoplasm, are largely unknown. Here we found that TIPE2 was highly expressed in the very low risk GIST tissues, and TIPE2 expression was gradually decreased in accordance with GIST risk grades. Nowadays, the most important independent prognostic factor for evaluating GIST risk grade is a high tumor mitotic rate [32, 35]. TIPE2 expression was negatively associated with tumor size and mitotic count, as well as the ratio of Ki-67 positive cells, which has been suggested as an alternative for mitosis counting [36]. Moreover, further analysis revealed that TIPE2 may serve as a promising biomarker for evaluating the risk grade of GIST.

Based on our IHC findings, we also extended the observations to investigate the effect of TIPE2 on the malignant behaviors of GIST cells. Likewise, TIPE2 acted as a tumor suppressor in GIST as well, for it suppressed the proliferation, colony formation, migration and invasion of GIST cells. Of note, TIPE2 effectively inhibited the invasiveness of GIST cells. Enhanced invasiveness usually leads to tumor metastasis, which is the main reason for the poor prognosis of GIST patients [32, 37]. Therefore, TIPE2 might represent a potential target for the treatment of GIST, and explicating the detailed mechanisms is indispensable.

Previous research found that TIPE2 functions through binding with Rac1 and subsequently inhibiting its activity [15, 17, 27, 38]. It has been reported that Rac1 signaling plays a pivotal role in the invasion and metastasis of GIST [39]. Nevertheless, the detailed roles of Rac1 in GIST are unclear. In the present study, the expression and roles of Rac1 in GIST was examined. It was found that Rac1 expression was gradually increased in accordance with GIST risk grades and negatively associated with that of TIPE2. Our further experiments demonstrated that Rac1 was also involved in the proliferation and invasiveness of GIST cells. Moreover, TIPE2 suppressed the proliferation and invasion of GIST cells via targeting Rac1.

TIPE2 has been demonstrated to promote the apoptosis by regulating caspase-9 and caspase-3 in both lung cancer and gastric cancer [40, 41]. TIPE2 also decreased the expression of uPA and MMP-9 in HCC via inhibiting the activity of Rac1 [15]. Here our results showed that TIPE2 induced the expression of caspase-9, caspase-3 and decreased MMP-9 expression in GIST cells. Several classical molecules that contributing to apoptosis and cell invasiveness have been demonstrated to be downstream effectors of Rac1 [20, 28]. Consistent with the aforementioned results, our data demonstrated that TIPE2 induced apoptosis and decreased MMP-9 expression of GIST cells in Rac1 dependent manner.

One of the greatest challenge for the treatment of GISTs in clinics is chemoresistance [1], the role of TIPE2 in the chemoresistance and its related mechanisms (such as its effect on cancer stem-like cells etc.) warrant further study. Moreover, the relationship between TIPE2 expression and the prognosis of GIST patients still needs to be further investigated. In addition, as the epitope for generating the antibody of TIPE2 was the same for both wild type TIPE2 and mutation TIPE2, it is difficult to detect the expression of mutant TIPE2 in GIST tissues.

In conclusion, the present study demonstrated that TIPE2 serve as a potential biomarker for evaluating the risk grade of GIST, which enriched the risk criteria for GIST. Moreover, by regulating tumor proliferation and invasiveness, forced TIPE2 expression may be considered as a potential therapeutic strategy in controlling the progression of GIST.

## Abbreviations

GISTs: gastrointestinal stromal tumors
TIPE2: tumor necrosis factor-alpha-induced protein 8-like 2
ICC: interstitial cells of Cajal
PDGFRA: platelet-derived growth factor receptor alpha
HCC: hepatocellular carcinoma
NSCLC: non-small cell lung cancer
MMPs: matrix metalloproteinase
uPA: urokinase plasminogen activator
FBS: fetal bovine serum
IHC: immunohistochemistry
ROC: receiver operating characteristic
AUC: area under the curve
NIH: National Institutes of Health
AFIP: Armed Forces Institute of Pathology
